# Quantitative sensory phenotyping in chronic neuropathic pain patients treated with unilateral L4-dorsal root ganglion stimulation

**DOI:** 10.1186/s12967-020-02566-8

**Published:** 2020-10-21

**Authors:** Thomas Kinfe, Nico von Willebrand, Andreas Stadlbauer, Michael Buchfelder, Thomas L. Yearwood, Sajjad Muhammad, Shafqat R. Chaudhry, Sascha Gravius, Thomas Randau, Klemens Winder, Christian Maihöfner, Nadine Gravius, Walter Magerl

**Affiliations:** 1 Division of Functional Neurosurgery and Stereotaxy, Department of Neurosurgery, Friedrich-Alexander University (FAU) Erlangen-Nürnberg, Schwabachanlage 6, 91054 Erlangen, Germany; 2 Department of Neurosurgery, Rheinische Friedrich-Wilhelms University, Bonn, Germany; 3 Department of Neurosurgery, Friedrich-Alexander University (FAU) Erlangen-Nürnberg, Erlangen, Germany; 4 Department of Pain Management, Guy’s and St Thomas’ Hospitals, London, UK; 5 Department of Neurosurgery, Heinrich-Heine University Düsseldorf, Düsseldorf, Germany; 6 Department of Basic Medical Sciences Shifa College of Pharmaceutical Sciences, Shifa Tameer-E-Millat University, Islamabad, Pakistan; 7 Department of Orthopedics and Trauma Surgery, University Hospital Mannheim, University Heidelberg, Heidelberg, Germany; 8 Department of Orthopedics and Trauma Surgery, Rheinische Friedrich-Wilhelms University Bonn, Bonn, Germany; 9 Department of Neurology, Friedrich-Alexander University (FAU) Erlangen-Nürnberg, Erlangen, Germany; 10 Department of Neurology, Hospital Fürth, Fürth, Germany; 11 Institute of Neurophysiology, Medical Faculty Mannheim, University Heidelberg, Heidelberg, Germany

**Keywords:** Dorsal root ganglion stimulation, Complex regional pain syndrome, Sensory quantitative testing, Objective measures for neurostimulation

## Abstract

**Background:**

In a previous study, we reported that selective dorsal root ganglion stimulation (DRG_STIM_) at DRG level L4 promoted a favorable outcome for complex regional pain syndrome (CRPS) patients along with DRG_STIM_-related changes of inflammatory biomarkers in blood and saliva. The impact on somatosensation is largely unknown. Herein, we assessed the quantitative sensory profile to quantify L4-DRG_STIM_ effects in CRPS patients.

**Methods:**

Twelve refractory CRPS patients (4 female; 8 male; mean age 69 ± 9 years) received standardized quantitative sensory testing (QST) protocol at baseline and after 3 months of unilateral L4-DRG_STIM_ assessing nociceptive and non-nociceptive thermal and mechanical sensitivity of the knee affected by CRPS and the contralateral non-painful knee area.

**Results:**

At baseline, CRPS subjects showed significantly increased thresholds for warmth, tactile and vibration detection (WDT, MDT and VDT) and exaggerated pain summation (WUR). After 3 months of unilateral L4-DRG_STIM_ all pain parameters exhibited trends towards normalization of sensitivity accumulating to a significant overall normalization for pain sensitivity (effect size: 0.91, p < 0.01), while with the one exception of WDT all non-nociceptive QST parameters remained unchanged. Overall change of non-nociceptive detection was negligible (effect size: 0.25, p > 0.40). Notably, reduction of pain summation (WUR) correlated significantly with pain reduction after 3 months of L4-DRG_STIM_.

**Conclusions:**

Selective L4-DRG_STIM_ lowered ongoing pain in CRPS patients and evoked significant normalization in the pain domain of the somatosensory profile. Thermoreception and mechanoreception remained unchanged. However, larger randomized, sham-controlled trials are highly warranted to shed more light on effects and mechanisms of dorsal root ganglion stimulation on quantitative sensory characteristics.

The study protocol was registered at the 15.11.2016 on German Register for Clinical Trials (DRKS ID 00011267).

https://www.drks.de/drks_web/navigate.do?navigationId=trial.HTML&TRIAL_ID=DRKS00011267

## Background

Targeted dorsal root ganglion stimulation (DRG_STIM_) achieved marked pain relief and improved the functional state of otherwise intractable chronic pain patients of different origin in randomized-controlled and observational in-human studies [1–3]. Previously published complex regional pain syndrome (CRPS) tonic spinal cord stimulation (SCS) trials reported a success rate between 40–50%, while randomized-controlled CRPS studies found an increased pain suppression rate using targeted sub-perceptional DRG_STIM_ varying between 50–70%. The mechanism of action of DRG-induced effects on spinal pain transmission remains largely unknown. DRG_STIM_ may inhibit hyperactive DRG neurons and deeper layer compartments of the spinal cord (dorsal column layers) relevant for neural pain transmission [1–3].

Most recently, we confirmed these reported findings in a cohort of complex regional pain syndrome (CRPS) patients treated with unilateral L4-DRG_STIM_ complying with the diagnostic criteria of CRPS. In addition, 3 months of adjunctive L4-DRG_STIM_ was found to alter levels of peripheral circulating mediators of inflammation in blood and saliva in favor of an anti-inflammatory state [4, 5]. Additional transcriptome analysis of the L4-DRG_STIM_ treated CRPS patients revealed a distinct upregulated—downregulated pattern of genes associated with inflammatory circuits pivotal for the development of CRPS (cytokine activity, glucose hemostasis, innate immune response, metabolic processing, sensory perception of pain, chronic inflammatory signaling, cell chemotaxis and neural synaptic transmission, cell chemotaxis, cell–cell signaling, vasodilatation, immune cell proliferation, cartilage development, cytokine synthesis, lipid metabolic function, angiogenesis, blood pressure and response to mechanical stimuli) [5]. Despite molecular inflammatory phenotyping, other outcome measures such as functional/structural neuroimaging or electrophysiological assessment by electroencephalography/laser evoked potential (EEG/LEP) means have been explored in the past as potential objective responsiveness parameters for spinal modulation therapy [4–8]. Notably, the determination of somatosensory thresholds has been used in order to objectively quantify SCS responsiveness in chronic pain patients albeit with conflicting findings. The predominantly applied SCS waveform represented conventional low-frequency SCS pattern, despite one study, in which high-frequency SCS (HFS) was utilized [9–17].

Neuropathic pain is characterized by a broad range of sensory dysfunction such as allodynia, hyperalgesia, hypoalgesia and hypoesthesia in need of an appropriately objective diagnostic tool, of which quantitative sensory testing (QST) is one [18–28]. Although CRPS-I has not been classified as a neuropathic pain per se in the most recent pain classification scheme, since except for CRPS-II the contribution of nerve damage cannot unequivocally assigned, many of the sensory features of CRPS are shared with neuropathic pain, in particular sensory loss and mechanical hyperalgesia/allodynia [29–32].

However, there are a few in-human studies, which use QST to quantify the effects of SCS effects on pain and sensory domains [6, 9, 12, 16, 17]. Our primary objective in this study was to determine the impact of selective, unilateral L4-DRG_STIM_ on quantitative sensory testing domains according to a standardized protocol in CRPS subjects treated with L4-DRG_STIM_, comparing both extremities (non-painful versus painful knee area).

## Methods

The QST study protocol represents a psychophysical analysis of our previously pilot study [5] addressed to molecular inflammatory phenotyping and was performed according to the guidelines of the latest revision of the declaration of Helsinki by authors NW and TK. An independent internal local ethical board/committee (IRB-No. 258/15) approved the study protocol and all patients provided informed consent.

The study was registered at the German Register for Clinical Trials (DRKS ID 00011267) (https://www.drks.de/drks_web/navigate.do?navigationId=trial.HTML&TRIAL_ID=DRKS00011267) on 15 Nov 2016.

### Data collection and characteristics of the study cohort at baseline

The study cohort consisted of 12 patients with CRPS resistant to conservative therapies, eligible for L4-DRG (mean age: 69 ± 9 years—age range: 50–80 years females and 4 males). Sleep quality was assessed using the Pittsburgh Sleep Quality Index (PSQI) [33]. Depressive symptom was evaluated by the Beck Depression Inventory (BDI) [34]. Mean body mass index (BMI) for the study cohort was 29.3 ± 5.6 kg/m^2^ (range: 23–40 kg/m^2^). Only three patients exhibited normal weight, five were classified pre-obese, obesity class I was present in one CRPS patient, obesity class II in two subjects and obesity class III in one patient. At least one or more of the following metabolic-associated disorders was present in all DRG subjects: hypertension, diabetes or cardiac ischemia. The average duration of conventional multimodal pain therapy was 5.2 ± 0.3 years.

### Selective L4 DRG-SCS implantation

The implant spine level was the DRG L4 with the following parameters: bipolar configuration, 20 Hz frequency, 200–300 µs pulse width, stimulation intensities 300–1600 µA applied over a period of 3 months. A single four-contact lead (Abbott Inc. Plano, Texas, USA) was implanted in the neuroforamina at DRG L4 on the ipsilateral side of the patient´s neuropathic pain. Adjustment of stimulation parameters were optimized to maintain satisfactory therapy in the sub-perception range throughout the entire 3-month study for each subject. The stimulation lead was externalized for each subject to allow a trial of stimulation lasting 7 days. Those patients achieving > 50% pain relief at the end of the trial period, compared to baseline, went on for permanent implantation of the pulse generator and completion of the study (3 months) [4, 5].

### Quantitative sensory testing (QST)

We employed Quantitative Sensory Testing (QST) in order to study distinct somatosensory profiles in each subject. We applied the standardized QST battery initially developed by the German Research Network on Neuropathic Pain (DFNS), which we have used in previous experimental and clinical studies and have been described in detail previously [25, 26, 35].

**Fig. 1:**
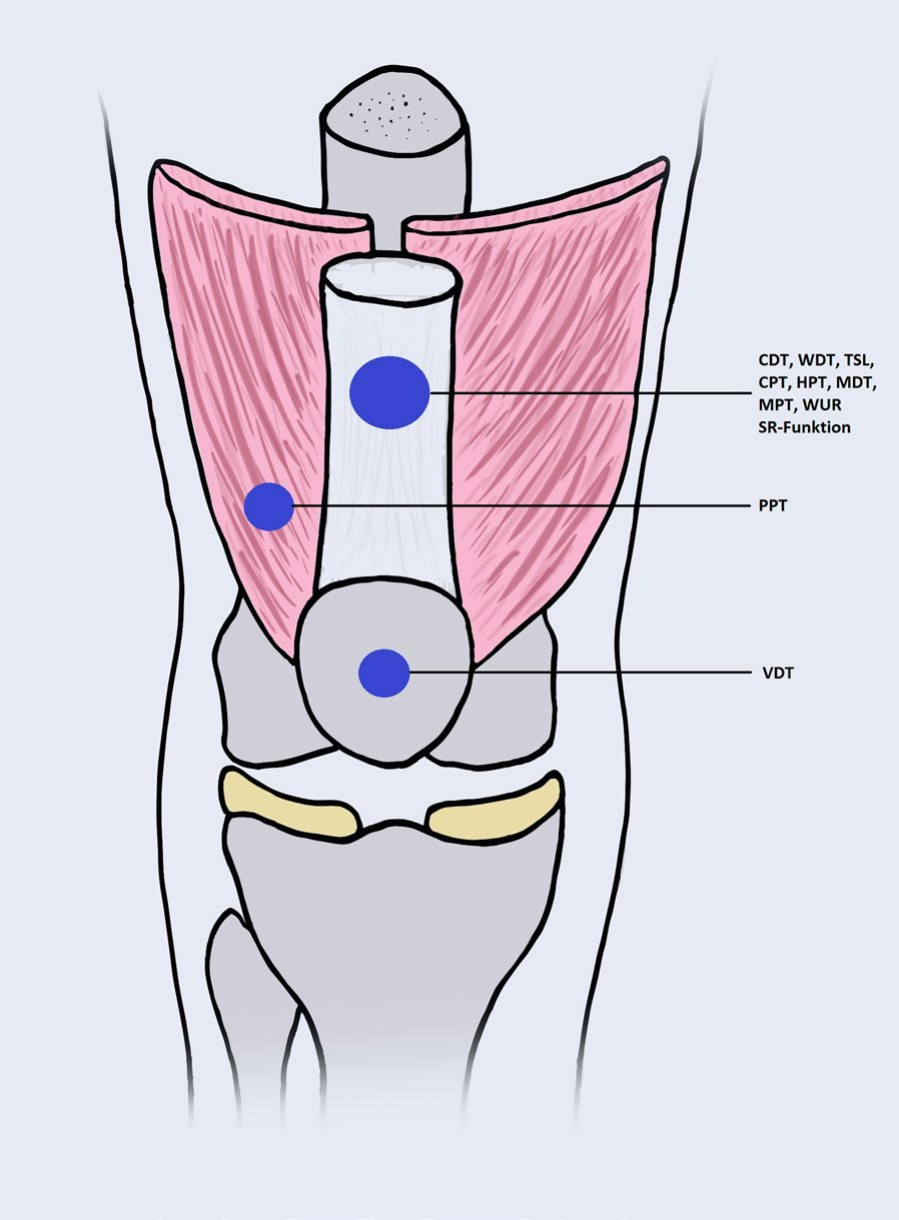
Schematic illustration of the anatomical knee region assessed by QST on both side, the affected and the non-affected knee

QST measurements was performed on a standardized time (09.00 a.m.) by the same investigators (TMK and NW) at baseline and follow-up and have been obtained from both knees, as shown in Fig. 1, to measure the following somatosensory test procedures (in the order of testing):

#### 1. Thermal detection and pain thresholds

Thermal detection and pain thresholds were investigated using a computer-controlled TSA 2001-II contact heat stimulator (Medoc® Inc., Israel) with a 3 × 3 cm area of a Peltier element and a 1 °C/s rate of rise from a baseline adaptation temperature of 32 °C and detection was signaled by the patient by pressing a button, which terminated the respective temperature change (method of limits). Thermal thresholds were determined in the following order: cold detection threshold (CDT), warmth detection threshold (WDT), alternating WDT/CDT, i.e. thermal sensory limen (TSL), cold pain threshold (CPT) and heat pain threshold (HPT). Mean threshold temperatures were calculated from three consecutive measurements, each. If cooling following a preceding warm stimulus in the TSL procedure was perceived as “not cool”, but warm, hot or burning this event was counted as the occurrence of paradoxical heat sensation (PHS) (Fig. 1).

#### 2. Mechanical detection thresholds

Modified “von Frey Filaments” were used for the measurement of mechanical detection thresholds (MDT). The von Frey filaments comprised a standardized set of stimulators which were calibrated to deliver forces between 0.25 and 512 mN in a geometric series with factor of 2 progression (Optihair 2, Marstock Nervtest, Germany). Threshold determinations were made with a series of ascending and descending stimulus intensities from five just suprathreshold and five just subthreshold forces (up-down adaptative threshold testing). The final threshold was the geometric mean of these ten values series returning a threshold at 50% hit rate (Fig. 1).

#### 3. Mechanical pain thresholds

Calibrated weighted pinprick stimuli were used for measurement of mechanical pain thresholds (MPT) with a set of seven pinprick mechanical stimulators with fixed stimulus intensities between 8 and 512 mN in a geometric series with factor of 2 progressions (up-down adaptative threshold testing). The criterion used to discriminate of sharp vs. non-sharp and the final threshold was the geometric mean of five series of ascending and descending stimuli (Fig. 1).

#### 4. Pain summation

Using the same technique as above, pain summation was tested by comparing pain ratings to a series of ten suprathreshold pinprick stimuli (mechanical pain sensation; MPS). Pain summation was tested by comparing pain ratings to a series of ten pinprick stimuli repeated at 1/s using a 256 mN pinprick vs. a single pinprick stimulus of the same force (wind-up ratio; WUR) (Fig. 1).

#### 5. Vibration detection thresholds

A 64 Hz tuning fork (Rydel-Seiffer 8/8 calibrated tuning fork) that was placed over the knee joint area in order to investigate vibration detection thresholds (VDT). The tuning fork was stroked and left in place until the subject could longer feel vibration. Vibration detection threshold (VDT) was determined as the average disappearance threshold with three stimulus repetitions (Fig. 1).

#### 6. Pressure pain threshold

Pressure pain threshold (PPT) was measured by a blunt pressure algometer (FDN200, Wagner Instruments, USA) over the knee joint area (area of contact 1 cm^2^). The stimulus force was ramped with a linear increase of 50 kPa/s (≈ 0.5 kg/s of weight loading) and the pressure pain threshold was determined with three series of ascending stimulus intensities (Fig. 1).

### Statistics

All data were analyzed by descriptive statistics and these raw data are presented as mean ± SEM. QST data in the affected knee area and the mirror-image control knee area at baseline, and after 3 months of continuous L4-DRG-stimulation are usually normalized to the DFNS reference data of healthy control subjects [35] in order to establish somatosensory profiles. Thus, all data are expressed as relative changes to a normal profile stratified for gender and age for the respective body region. This expresses the data in units of standard deviations of the reference data (standard normalized z-values). In this way, it is possible to identify sensory deviations from reference data at baseline, of side-to-side differences, and of treatment-related changes at follow-up in the same dimensionless scaling for all sensory parameters. The calculation of standard normal distributions (Z-transformation) has been established as a valid method in previous QST studies on experimentally-induced sensory changes [22, 38], patient profiling in e.g. neuropathic pain, neuroinflammatory pain, and inflammatory bowel diseases [14], in CRPS [29–32, 36], and as an endpoint in stimulation-related treatment studies [13, 37]. Since no reference data exist for the knee joint area [closest site for reference being the foot dorsum) we used the contralateral non-painful knee to express the site-specific sensory changes in the CRPS-affected knee.

Z-transformation value is a standard procedure in QST- analysis within the German Research Network on Neuropathic Pain (DFNS) c.f. [35]. We calculated Z-values as follows:

$$Z = ({\text{individual value}} - {\text{mean}}_{{{\text{control}}\,{\text{site}}}} )/{\text{SD}}_{{{\text{control}}\,{\text{site}}}}$$

All QST data obtained in this study comparing the differences between baseline and post-stimulation in both the painful and the contralateral (control) knee were subjected to”Cohens d” to determine the size effect of any changes (https://www.socscistatistics.com/effectsize/default3.aspx).

## Results

### Skin temperature and spontaneous pain

**Fig. 2:**
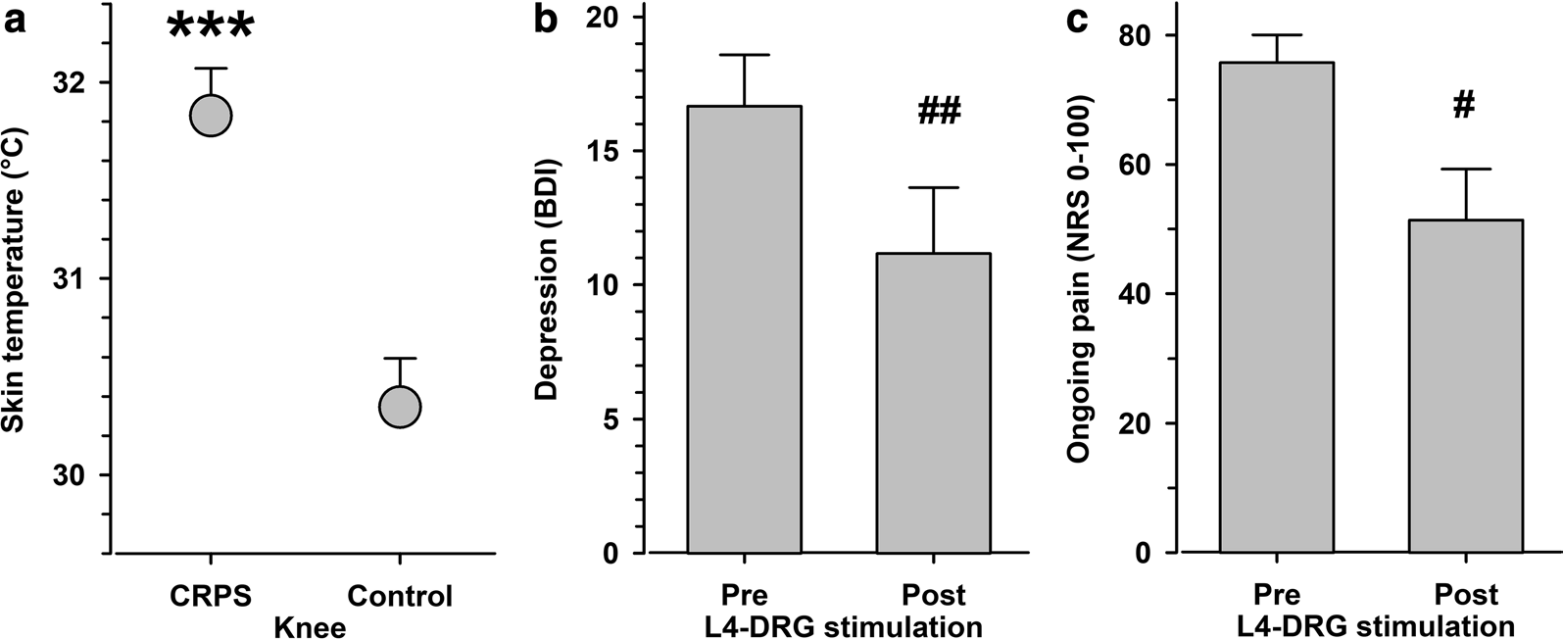
**a** Skin temperature in the affected and controlateral control knee. **b** Levels of depression on the Beck Depression Inventory (BDI) before and after L4-DRG stimulation. **c** Ongoing pain in the knee affected by CRPS before and after L4-DRG stimulation. ***p <  < 0.0001 CRPS-affected vs. contralateral control knee, ^#^p < 0.05, ^##^p < 0.01 pre vs. post L4-DRG stimulation

For all QST measurements, room temperature was kept at an ideal ambient level (24.3 ± 0.3 °C, mean ± SEM) and did not differ between baseline testing and follow-up (p = 0.66). Accordingly, all patients were in a neutral thermoregulatory state with an average skin temperature of 30.3 ± 0.2 °C (range 28.0–32.2 °C). Skin temperature was significantly higher in the pain-affected skin area with 31.8 ± 0.2 °C (range 29.0–34.0 °C, p < 0.001 at baseline and at follow-up). Notably, at individual inspection, all patients were identified as the warm CRPS subtype and a history of neuropathic pain of 5.2 ± 0.3 years [29, 38]. Temperature asymmetry at baseline did not change at follow-up (1.4 ± 0.3 vs.1.6 ± 0.3 °C; p = 0.62, Fig. 2a).

Patients exhibited substantial depression scores on the BDI (16.7 ± 1.9), which reduced significantly at 3 months follow-up after L4-DRG stimulation (11.2 ± 2.5; p < 0.01; Fig. 2b). While 6/12 patients exhibited clinically relevant levels of depression at baseline, this reduced to 2/12 (chi-square p < 0.10). Moreover, patients exhibited substantial sleep problems as assessed by the Pittsburgh Sleep Quality Index (PSQI 10.9 ± 5.6) with 9 patients exceeding the 5 point cut-off of normal sleepers, which reduced only marginally at follow-up (PSQI 8.8 ± 4.7; p = 011; 6 patients exceeding the 5 point cut-off).

Levels of spontaneous pain at baseline were 76 ± 4 on a 0–100 NRS. Following L4-DRG-stimulation, pain levels at 3 months follow-up diminished to 51 ± 8 NRS on average (p < 0.05), representing 30 ± 11% of pain reduction (Fig. 2c). This corresponds to a large effect size of 1.1095 (Cohen’s d). At single patient level, eight patients reported reduced pain, three patients the same level of pain and only one patient reported a modest pain increase. There was no difference in pain relieve between male and female patients (− 39 ± 21 vs. − 25 ± 13%, p = 0.58; Fig. 2c).

### Non-nociceptive somatosensory detection thresholds

**Fig. 3:**
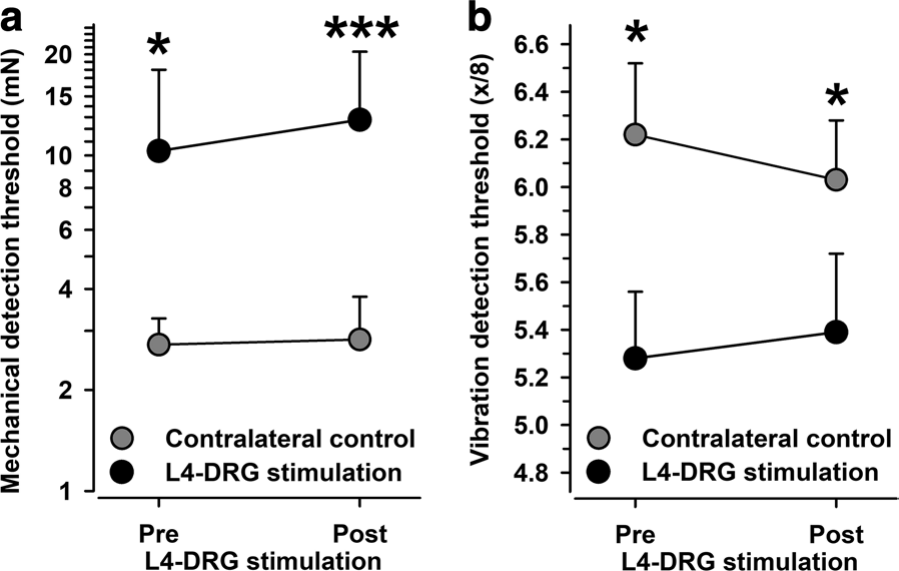
Mechanical detection thresholds (MDT) (**a**) and vibration detection thresholds (VDT) (**b**) in the CRPS-affected knee (black circles) and in the contralateral control knee (grey circles). Both mechanoreceptive parameters were significantly higher in the affected knee, which did not change after L4-DRG stimulation. *p < 0.05, ***p < 0.001 vs. contralateral control knee

Tactile detection thresholds (MDT) reflecting slowly adapting mechanoreceptor thresholds and flutter/vibration thresholds (VDT) reflecting rapidly adapting mechanoreceptor thresholds were significantly enhanced at baseline (both at least p < 0.02 vs. unaffected contralateral control side; Fig. 3). While tactile thresholds assessed by von Frey hairs were in the normal range of age-and gender-matched reference values in the unaffected control side (2.7 mN; c.f.), they were fourfold higher in the affected skin area (10.3 mN, p < 0.02). This ratio remained completely unaltered at follow-up (2.8 vs. 12.8 mN, p < 0.001 vs. control; both test areas, p > 0.50 vs. baseline testing; effect size: Cohen’s d = 0.2648).

At baseline, flutter/vibration sensitivity as tested with an 8/8 calibrated Rydel-Seiffer tuning fork (VDT) was significantly reduced compared to the control side (5.28 ± 0.28 vs. 6.22 ± 0.30 units, p < 0.05), which remained unchanged at follow-up in both test areas (5.39 ± 0.33 vs. 6.03 ± 0.25 units, p < 0.05; both test areas, p > 0.40 vs. baseline testing; effect size: Cohen’s d = 0.1360). Global impact on mechanoreception comprising MDT and VDT (compound factor “tactile sensitivity”) confirming the small effect size (Cohen’s d = 0.2817).

**Fig. 4:**
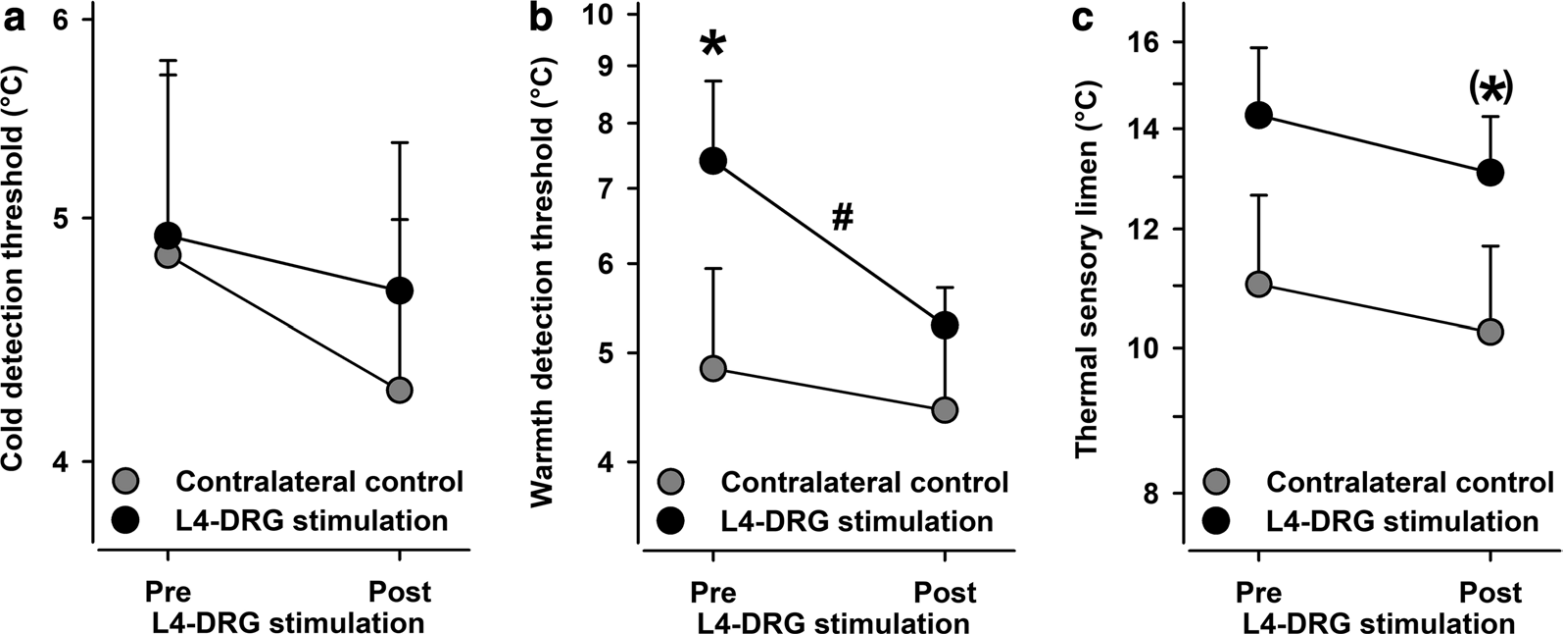
Thermal detection thresholds. **a** Cold detection thresholds (CDT) did not differ before or after L4-DRG stimulation. **b** Warmth detection thresholds (WDT) were significantly higher in the CRPS-affected knee (black circles) than in the contralateral control knee (grey circles) and normalized significantly after L4-DRG stimulation. **c** Thermal sensory limen (TSL) was higher in the affected knee. *p < 0.05, (*)p < 0.10 vs. contralateral control knee, #p < 0.05 pre vs. post L4-DRG stimulation

Cold detection (CDT; Fig. 4a) remained unchanged (effect sizes for CDT: Cohen’s d = 0.1638, and TSL: Cohen’s d = 0.0160). Warmth detection thresholds (WDT) were in the normal range of age-and gender-matched reference values in the unaffected control side (Fig. 4b). However, they were significantly enhanced in the affected area at baseline (7.4 vs. 4.8 °C, p < 0.02 vs. unaffected contralateral control side; Fig. 4b), which normalized significantly following L4-DRG-stimulation (5.3 vs. 7.4 °C, p < 0.05 follow-up vs. baseline). However, the effect size was modest (Cohen’s d = 0.4333) and given that a weak improvement of warmth sensitivity at follow-up also occurred in the control area the normalized difference failed to be significant (p = 0.20). Likewise, thermal sensory limen (TSL Fig. 4c) remained unchanged (effect sizes for CDT: Cohen’s d = 0.1638, and TSL: Cohen’s d = 0.0160). All thermal detection parameters aggregated in one compound factor (“thermal detection”) revealed an absence of significant change (effect size: Cohen’s d = 0.0921).

### Cold and heat pain thresholds

**Fig. 5:**
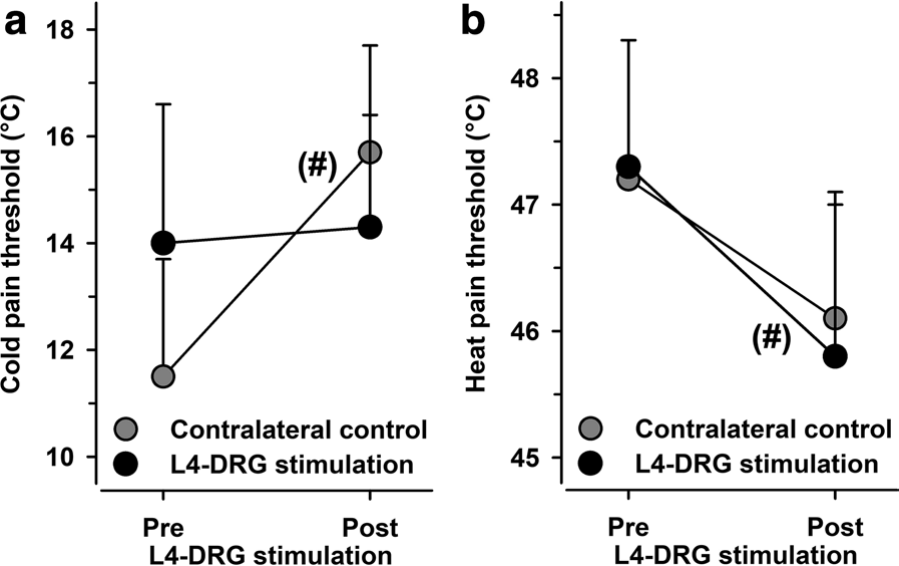
Thermal pain thresholds. Cold pain thresholds (CPT) (**a**) and heat pain thresholds (HPT) (**b**) in the CRPS-affected side did not differ from the control sites either before or after L4-DRG stimulation. (#)p < 0.10 pre vs. post L4-DRG stimulation

Cold pain thresholds (CPT) and heat pain thresholds (HPT) were in the normal range of age-and gender-matched reference values in the unaffected control side. Patients were marginally more cold pain sensitive, but cold pain thresholds did neither change in the control area (11.5 ± 2.2 vs. 15.7 ± 2.0 °C, n.s.) nor in the affected area (14.0 ± 2.6 vs. 14.3 ± 2.1 °C, n.s.; effect size: Cohen’s d = 0.4807) following L4-DRG-stimulation (Fig. 5). The same was observed for heat pain thresholds (control side: 47.3 ± 1.0 vs. 45.8 ± 1.2 °C, n.s.; affected side: 47.2 ± 1.1 vs. 46.1 ± 1.0 °C, n.s.; effect size: Cohen’s d = 0.1102). Both thermal pain parameters aggregated in one compound factor (“thermal pain”) confirmed the detailed findings (effect size: Cohen’s d = 0.3675).

### Pressure pain thresholds and pain summation

**Fig. 6:**
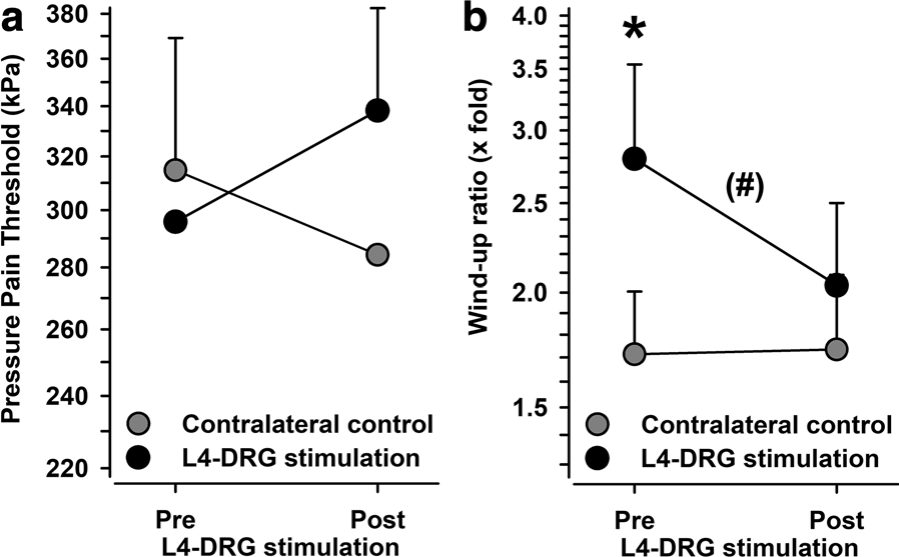
Pressure pain thresholds (PPT) (**A**) and pain summation (windup ratio WUR) (**B**) in the CRPS-affected knee (black circles) and in the contralateral control knee (grey circles). Pain summation was significantly higher in the affected knee, with a trend towards normalization after L4-DRG stimulation. *p < 0.05 vs. contralateral control knee, (#)p < 0.10 pre vs. post L4-DRG stimulation

Analysis of pain threshold to blunt pressure (PPT) revealed a similar trend (Fig. 6). Although changes between baseline and follow-up were not significant in both test areas, there was a reciprocal change of pressure pain thresholds following L4-DRG-stimulation. While pressure pain thresholds lowered in the control area they increased in the affected area combining to a significant trend in the normalized data (p < 0.10). The effect size of this change appeared to be medium to large (Cohen’s d = 0.6639).

Finally, pain summation upon repeated pinprick stimulation (WUR) was significantly enhanced in the affected area at baseline (2.79- vs. 1.71-fold increase, p < 0.05 vs. unaffected contralateral control side). While pain summation remained unchanged in the control area (1.73- vs. 1.71-fold increase, p > 0.90 vs. baseline), it tended to normalize in the affected area following L4-DRG-stimulation (2.04- vs. 2.79-fold increase, p < 0.10 vs. baseline). This effect remained on the level of a significant trend also in the normalized data (p < 0.10), although the effect size was only small to medium-sized (Cohen’s d = 0.4529). Notably, the magnitude of pain summation in the affected area but not in the control area correlated negatively with the subsequent reduction of spontaneous knee pain (r = −  0.56, p < 0.05). In addition, the reduction of pain summation correlated positively with the reduction of spontaneous knee pain (r =  + 0.49, p < 0.05).

Since we have recently found that principal component analysis revealed a communality of pressure pain threshold (PPT) and pain summation (WUR) [39] we added a tentative analysis of the combined normalized data. This analysis revealed an increased pain sensitivity at baseline although it failed to be significant (+ 0.45 ± 0.26 z-values, p = 0.12). This compound pain parameter normalized completely following L4-DRG-stimulation (−  0.06 ± 0.21 z-values at follow-up, p < 0.05 vs. baseline and p = 0.81 vs. control side). In this compound pain parameter, the magnitude at baseline in the affected area but not in the control area correlated negatively with the reduction of spontaneous knee pain (r = −  0.49, p < 0.05). The effect size of reduction towards normal values appeared to be medium to large (Cohen’s d = 0.5911). Moreover, the magnitude of normalization correlated positively with the reduction of spontaneous knee pain (r =  + 0.46, p = 0.058).

As the original principal component encompassed CPT, HPT, PPT and WUR [39] we also calculated the compound changes although analysis of thermal pain data alone did not yield significant results. This approach is further supported by the high correlation of the L4-DRG-stimulation-induced changes in the PPT/WUR and CPT/HPT subcomponents (r = 0.69, p < 0.005). Analysis of the resulting superfactor (“peripheral nociception”) returned the same findings as described above (effect size Cohen’s d = 0.5609) and further improved the strength of correlations with spontaneous knee pain reduction to r = −  0.58 and r = 0.56 (p < 0.05, each).

### Mechanical pain thresholds and pain ratings

**Fig. 7:**
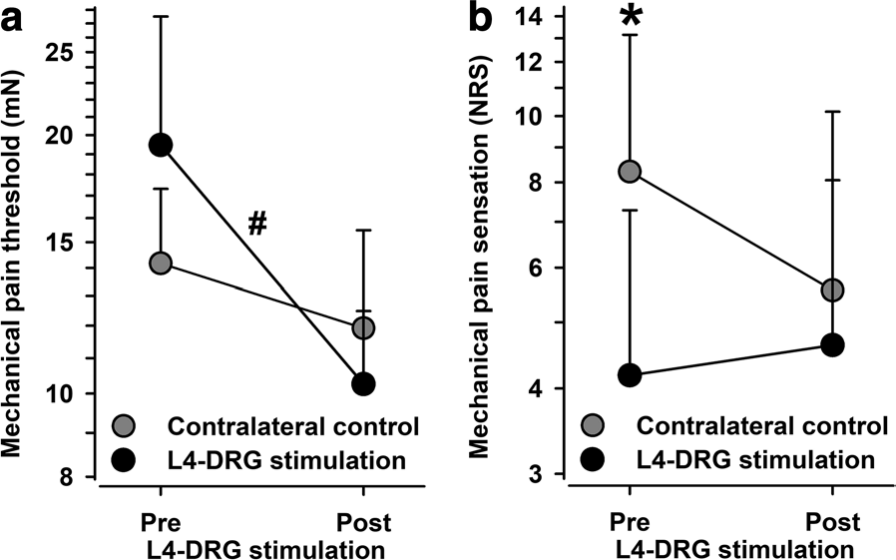
Mechanical pain thresholds (MPT) (**a**) and mechanical pain sensation (MPS) (**b**) in the CRPS-affected knee (black circles) and in the contralateral control knee (grey circles). Both parameters reflecting pain to punctate stimuli (pinprick) disclosed lower sensitivity in the affected knee, which normalized to the level of the control knee after L4-DRG stimulation. *p < 0.05 vs. contralateral control knee; #p < 0.05 pre vs. post L4-DRG stimulation

Mechanical pain sensitivity appeared to be improved consistently following L4-DRG-stimulation (Fig. 7). Although not statistically significant, there was a higher pain threshold to punctate mechanical stimuli (MPT) in the affected skin (19.5 vs. 14.2 mN, n.s.), which lowered significantly following L4-DRG-stimulation (19.5 vs. 10.3 mN, p < 0.02). Although there was also some (non-significant) increase in pinprick sensitivity in the control side (thresholds 11.9 vs. 14.2 mN, n.s.), there remained a significant trend in the normalized data (p < 0.10), the effect size of which appeared to be small to medium-sized (Cohen’s d = 0. 4688).

Analysis of pain ratings to supra-threshold pinprick stimuli (MPS) supported this finding. In the affected area, pain ratings to pinprick stimuli (4.2 vs. 8.6 NRS, p < 0.05). Although, in neither test area pain ratings changed significantly, pain ratings increased in the affected area, which became significant in the normalized pain ratings (p < 0.02), a small to medium-sized effect (Cohen’s d = 0.3160).

MPT and MPS are aspects of the same somatosensory modalities hence we added an analysis of the combined normalized data. This analysis revealed again the finding of a reduced pinprick sensitivity at baseline. Although, the reduction of pinprick sensitivity was not significant at baseline (−  0.44 ± 0.37 z-values, p = 0.25) it normalized completely following L4-DRG-stimulation (+ 0.04 ± 0.20 z-values at follow-up, p < 0.05 vs. baseline sensitivity, effect size: Cohen’s d = 0.4654) and it did not differ from control values after L4-DRG stimulation (p = 0.85 vs. control side).

### Normalization of pain-associated QST vs. non-pain-associated QST

**Fig. 8:**
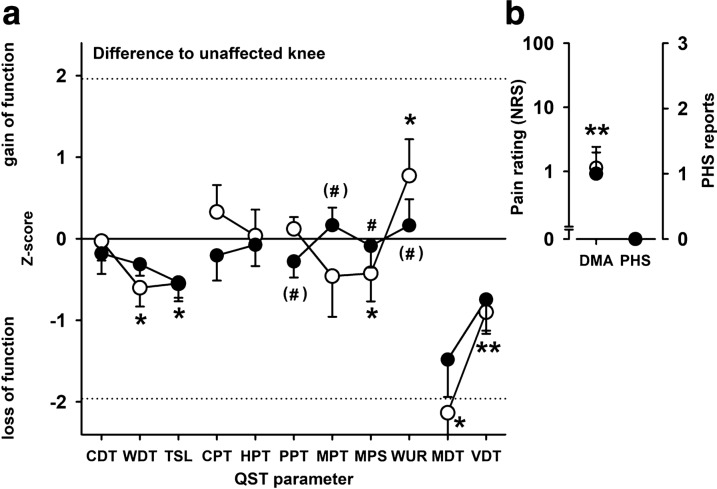
Somatosensory profile of the CRPS-affected knee before (open circles) and after L4-DRG stimulation (black circles) normalized to QST from the contralateral control knee (**a**) and mechanical or thermal dysesthesias (DMA, PHS) (**b**). Sensitivity reduced after L4-DRG stimulation for cold pain threshold (CPT), pressure pain threshold (PPT) and pain summation (WUR). Sensitivity increased after L4-DRG stimulation for warmth detection threshold (WDT), mechanical pain threshold (MPT) and mechanical pain threshold (MPS). **p < 0.01, *p < 0.05, (*)p < 0.10 vs. contralateral control knee; #p < 0.05, (#)p < 0.10 pre vs. post L4-DRG stimulation

**Fig. 9:**
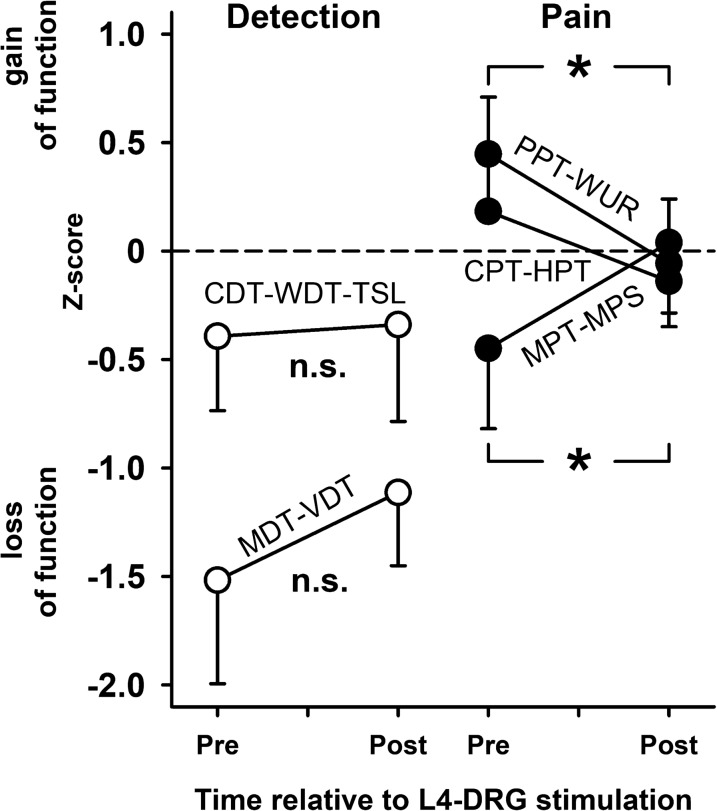
Aggregated somatosensory parameters (somatosensory principal components) in the CRPS-affected knee before (“Pre”) and after L4-DRG stimulation (“Post”) normalized to QST from the contralateral control knee. Sensitivity normalized for all pain components, but not for thermal and mechanical detection. Pressure pain/pain summation (PPT/WUR), thermal pain (CPT/HPT), punctate mechanical pain (MPT/MPS), thermal detection (CDT/WDT/TSL) and mechanical detection (MDT/VDT). Open circles—Non-nociceptive components “(detection”); Black circles—Nociceptive components (“pain”). *p < 0.05 pre vs. post L4-DRG stimulation.

Comparing all the changes in the QST profile revealed that there were non-significant trends in any of the pain-associated QST parameters towards side differences occurring prior to DRG stimulation and a general trend in most of these parameters to reestablish somatosensory symmetry between the CRPS-affected and the unaffected control knee (Fig. 8). This is even more obvious when the individual parameters were aggregated into higher order compound parameters for thermal pain, punctate mechanical pain and pressure pain/pain summation (Fig. 9) returning significant normalization for the two compound parameters of mechanical pain (p < 0.05, each; see also above). Although far from significant, the changes of thermal pain followed this trend.

Contrary, the non-nociceptive mechanoreceptive or thermoreceptive compound parameters normalized only to a very small extent (both p > 0.20) and mechanoreception and thermoreception in the CRPS-affected knee remained significantly less sensitive than in the control knee test site (p < 0.05 and p < 0.01, respectively; Fig. 9). Based on these patterns we contrasted the normalization of pain-associated QST to non-pain-associated QST by aggregating them into two overall grand average parameters. Non-nociceptive detection was significantly different from the control knee (p < 0.005) and remained so after L4-DRG stimulation (p < 0.05) and normalization to symmetry was very small (effect size: Cohen’s d = 0.2593, p = 0.41). In contrast, L4-DRG stimulation normalized pain-associated QST significantly with a large effect size (Cohen’s d = 0.6770, p < 0.01) being significantly different from the unaffected control knee before, but not anymore after DRG-L4 stimulation (p > 0.60).

Reduction of ongoing pain correlated significantly with the normalization of pain QST (r = 0.51, p < 0.05). An association of CRPS ongoing pain reduction with normalization of the pain associated QST was fostered by median split analysis. Stratification of the patients by the magnitude of pain relieve (either less or more than 30% of pain reduction according to the Farrar criterion of clinically relevant pain reduction) [40] revealed that the high relieve cohort (n = 6) reported a substantial average pain relieve (−  60 ± 11%, p < 0.005), while the low relieve cohort (n = 6) reported no pain relieve at all (+ 1 ± 5%, p > 0.90). Normalization of pain-related QST was significantly different between both cohorts (p < 0.05), namely 0.677 ± 0.204 z-values towards normal sensitivity in the high relieve cohort (p < 0.02), but not in the low relieve cohort (0.194 ± 0.140, p > 0.20). In contrast, normalization of detection did not discriminate both cohorts (p = 0.30).

### Levels of circulating inflammatory at baseline and after 3 months of adjunctive, unilateral L4-DRG stimulation

Previous findngs from our L4-DRG Stim—neuroinflammation cytokine study indicated that CRPS subjects displayed a pro-inflammatory profile as an expression of an ongoing disease state although we found improvement in pain intensity quantified by sensory testing in our current report. Briefly, we found the following changes before and after L4 DRG assessing concentrations of inflammatory makers by immunoassay means [4].

**Fig. 10:**
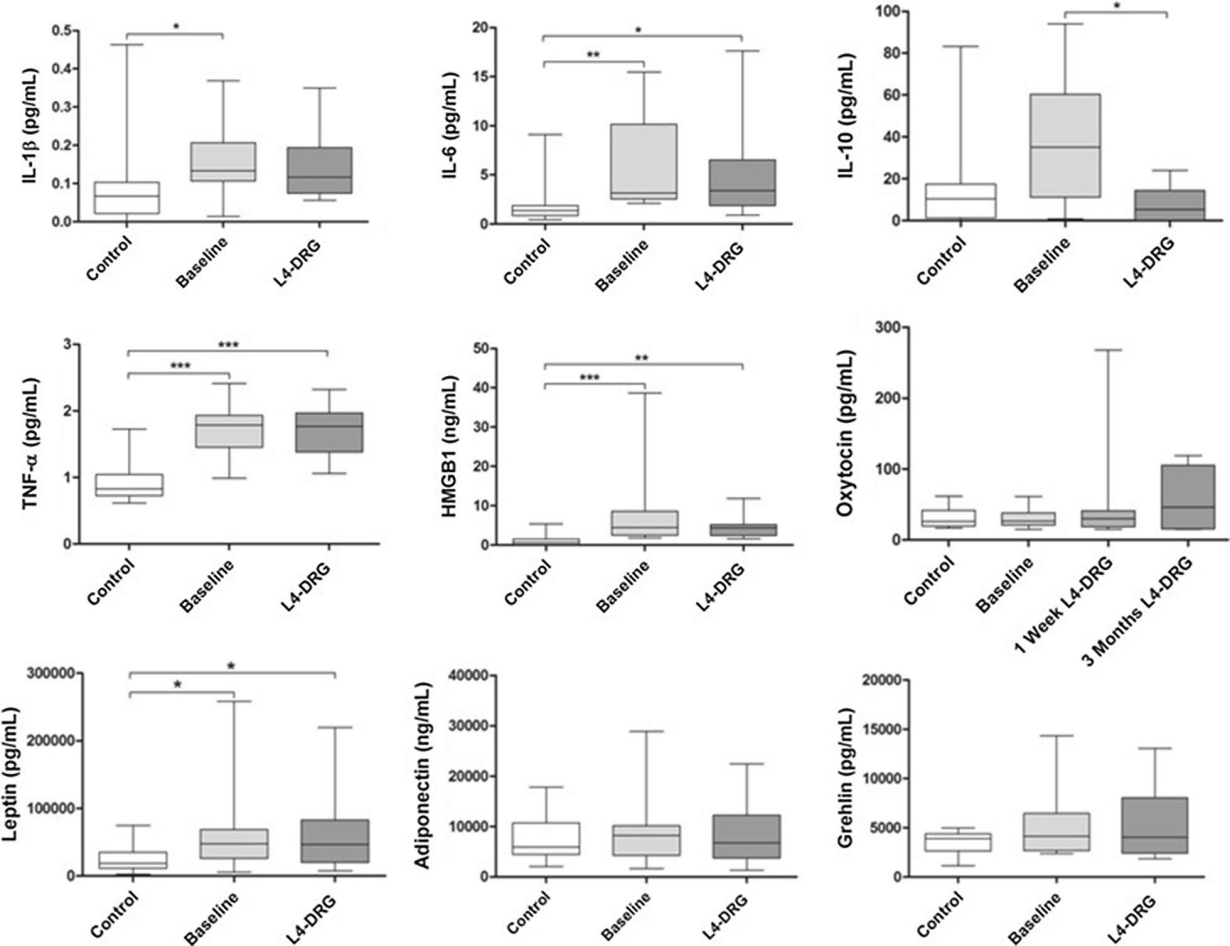
Interleukin-1ß (IL-1 ß), interleukin 6 (IL-6), tumor necrosis factor (TNF-α), high-mobility group box 1 protein (HMGB1), interleukin-10 (IL-10), leptin, adiponectin and ghrelin serum immunoassays. A comparison of baseline assessment and after 3 months selective L4-DRG_STIM_ (two right columns) compared to those of healthy controls (HC). Saliva concentrations of oxytocin at baseline, after 1 week L4-DRG_STIM_ trial and after 3 months. A comparison of baseline assessment and after 3 months selective L4-DRG_STIM_ (two right columns) compared to those of healthy controls (HC). For saliva oxytocin an additional measure was performed after 1 week trial stimulation. Mean values with standard deviation and p-values. ^*/**/***^ indicates p-values < 0.05 (statistically significant) [4]

Significantly elevated levels were found before and after 3 months L4-DRG_STIM_ compared to healthy controls (HC) for HMGB-1 (HC: 1.2 ± 1.6 ng/ml versus pre-DRG_STIM_: 7.7 ± 10.14 ng/ml versus post-DRG_STIM_ 4.3 ± 2.7 ng/ml; p = 0.0001), TNF-α (HC: 0.94 ± 0.3 pg/ml versus pre-DRG_STIM_: 1.72 ± 0.39 pg/ml versus post-DRG_STIM_: 1.71 ± 0.4 pg/ml; p = 0.0001), IL-6 (HC: 2.14 ± 2.47 pg/ml versus pre-DRG_STIM_: 5.61 ± 4.85 pg/ml versus post-DRG_STIM_: 5.54 ± 5.6 pg/ml; p = 0.0008) and leptin (HC: 23,666 ± 17,828.5 pg/ml versus pre-DRG_STIM_: 65,758.33 ± 69,321.69 pg/ml versus post-DRG_STIM_: 60,975 ± 58,537.67 pg/ml; p = 0.015). Serum concentration of IL-1b was significantly elevated at baseline compared to healthy controls (HC: 0.09 ± 0.1 pg/ml versus pre-DRG_STIM_: 0.16 ± 0.1 pg/ml; p = 0.0178), but not after 3 months L4-DRG_STIM_ (0.14 ± 0.1 pg/ml). BDNF serum levels were higher in CRPS subjects and remained unchanged after L4-DRG_STIM_ (HC: 31,424,18 ± 9326,80 pg/ml versus pre-DRG_STIM_: 39,425.40 ± 10,234.85 pg/ml versus post-DRG_STIM_: 38,699.21 ± 8054.56 pg/ml). Markers of immunometabolic signalling such as adiponectin (HC: 7391.67 ± 4144.78 pg/ml versus pre-DRG_STIM_: 8612.50 ± 7063.3 pg/ml versus post-DRG_STIM_: 8681.67 ± 6603.1 pg/ml) and ghrelin (HC: 3538.5 ± 1065.95 pg/ml versus pre-DRG_STIM_: 5307.5 ± 3715. 6 pg/ml versus post-DRG_STIM_: 5464.6 ± 3842.9 pg/ml) remained unchanged between controls, pre- and post L4-DRG_STIM_ CRPS subjects. Increased IL-10 serum concentrations were detected at baseline compared to healthy subjects and significantly decreased after 3 months L4-DRG treatment (HC: 13.78 ± 19.1 pg/ml versus pre-DRG_STIM_: 38.06 ± 29.71 pg/ml versus post-DRG_STIM_: 7.61 ± 8.12 pg/ml; p = 0.0063). Saliva oxytocin levels were higher in CRPS patients compared to HC and increased after 1 week of L4-DRG_STIM_ (trial phase) and after 3 months L4-DRG_STIM_ (HC: 30.45 ± 14.38 pg/ml versus pre–DRG_STIM_: 32.58 ± 13.0 pg/ml versus post-DRG_STIM_ 1 week: 55.35 ± 75.01 pg/ml versus post DRG_STIM_ 3 months: 59.82 ± 41.89 pg/ml; p = 0.65) (Fig. 10). C-reactive protein (CRP) values assessed within the study period were low (average 0.34—0.48 mg/dl) [4].

### Complications associated with implantation and/or stimulation

No complications related to the stimulation or the implantation procedure occurred within the study period.

## Discussion

### Brief summary of study findings and comparison with published in-human clinical data

The sensory profile of CRPS patients was in line with previous reports, in particular when data were compared to the warm CRPS subtype rather than to an unselected cohort. This encompassed mechanoreceptive and thermoreceptive sensory loss, which was less prominent than reported in the cold CRPS subtype, as well as in neuropathic pain [29–32, 36, 38, 40, 41]. This was in line with the absence of paradoxical heat sensation in chronic CRPS [31, 32, this study] for which the expression was related to the magnitude of sensory loss [40]. Conspicuously, hyperalgesia to blunt pressure as well as pinprick was less prominent than reported previously for distal extremities [30, 32]. We have recently shown that pressure hyperalgesia is likely precipitated indirectly by reduced movement as a disuse-related sensory alteration [32]. Likewise, physical therapy and occupational therapy is an integral element of CRPS therapy [42]. Since the knee joint is such an integral part in body movement this may explain, why in this particular cohort pressure hyperalgesia is virtually absent. In contrast, the majority of patients reported bilateral pain to light touch (dynamic mechanical allodynia), which is a hallmark sign of central sensitization in human surrogate models as well as in pain patients [12, 40, 43–45].

After 3 months L4-DRG stimulation, patients reported a significant reduction of ongoing pain and normalization of nociceptive QST regardless of the direction of deviation from the control side. The nociceptive parameter exhibiting the most pronounced normalization upon L4-DRG stimulation comprised the lowering of pressure pain sensitivity and of temporal summation of pain (“wind-up”). Interestingly, a recent SCS study also reported the normalization of temporal summation and reduction of pressure pain sensitivity in the ON condition of SCS, the latter interpreted as restoration of endogenous pain control [46]. This may be of particular importance, since the synergy of exaggerated temporal summation and absence of endogenous pain control is currently hypothesized to be predictive of enhanced risk of chronic pain by altering the balance of central pro- vs. antinoceptive mechanism [28, 47, 48] for which QST is a validated methods for assessment of patient profile and a tool in drug discovery [3, 28]. In chronic CRPS some of the sensory dysfunction is often mirrored in the unaffected, contralateral anatomical location [31, 32]. Thus, the sensory changes reported in this study may be underestimated.

L4-DRG stimulation was accompanied by a significant reduction of patients’ level of depression. The latter is in line with previous reports and meta-analyses suggesting implantable devices lead to significant mood improvements [49]. It has been repeatedly reported that emotional dysregulation is unexpectedly severe in CRPS with high levels of anxiety and depression, and of posttraumatic stress disorder [29, 32, 50]. Notably, although successful treatment with implantable devices lead improvement, high levels of depression in particular has been associated as negative predictors of neurostimulation success [51–54].

Although we found a meaningful clinical response and improved QST parameters, with regard to circulating inflammatory mediators assessed in our previously published study, increased serum values of pro-inflammatory markers have been quantified pre- and post L4-DRG_STIM_ compared to healthy controls for HMGB-1, TNF-α, IL-6 and leptin. IL-1β was significantly elevated pre-L4 DRG_STIM_, but not post-treatment indicating an ongoing persistent pain state. Elevated anti-inflammatory IL-10 significantly decreased after 3 months in serum, while saliva oxytocin concentrations increased in CRPS subjects after L4-DRG_STIM_ [4].

### Comparison with published human clinical SCS trials assessing somatosensory perception

There exist a number of uncontrolled and randomized-controlled studies assessing possible associations between spinal cord stimulation and sensorimotor detection thresholds and pain perception. Comparative conclusions appear to be limited due to the heterogeneous study protocols and pain disorders investigated. Firstly, Kemler and colleagues investigated a homogenous study cohort consisting of CRPS1 patient randomized to SCS plus physical therapy versus CRPS 1 subjects only receiving physical therapy [10, 11].

Repetitive QST measurements performed over a period of 12 months demonstrated no differences in all QST detection thresholds when comparing both groups indicating that SCS appear to have no effects on the sensorimotor profile of CRPS patients, although SCS reduced pain levels and improved health-related quality of life. These findings were contrary to those results reported from Lindblom’s and Meyerson [55] but were confirmed by Troels Jensen’s group in a later SCS trial under randomized-controlled conditions in a smaller number of CRPS subjects [12]. Importantly, the improvements reported in the present study were all of borderline significance as individual estimates. Robust normalization was identified by aggregating individual parameters to higher order principal components, which revealed that improvement only occurred in pain related QST, but not in mere sensory detection. Another observational prospective trial evaluated the impact of SCS on QST domains under SCS “off” condition in a relatively small cohort of patients (solely male patients) suffering from unilateral radicular neuropathic pain resulting from failed-back surgery syndrome (FBSS). Notably, duration between inactive and active SCS ranged between 30 and 60 min in contrast to the extended observation period provided by the study of Kemler and co-workers [10, 11, 16].

Under inactive SCS significantly increased thresholds for tactile and warm/cold detection thresholds have been observed at baseline, which is in line with the data we report, while with active SCS cold/warm and mechanical detection thresholds significantly reduced compared to the contralateral, non-painful extremity [16], a finding that we and also other studies in chronic pain disorder cohorts of various origin could not confirm [56].

SCS was reported to normalize temporal summation and to restore conditioned pain modulation, i.e. dynamic pain inhibition [46]. Interestingly, it has been shown that clinical neurophysiological parameters obtained from the lower extremities, like the P40-SEP, H-reflex and nociceptive RIII reflex were lowered in the “on” condition of the SCS stimulator. Pain reduction and reduction of the nociceptive RIII reflex were correlated pointing to a spinal site of action [57]. Extending the application of QST towards primary Raynaud’s syndrome, lumbar and thoracic tonic SCS waveforms (active versus inactive SCS) was found to significantly modulate sensory properties including cold detection, mechanical detection, mechanical pain detection and vibration thresholds in one RS patient compared to healthy subject data [15].

According to the published QST studies assessing possible relationships between tonic SCS waveform and sensorimotor phenotyping, Bordeleau and colleagues concluded, that it is more likely that tonic SCS did not interfere with perception of external stimuli (experimental pain) due to the broad range of pain disorders included, different applied methodologies of QST (different devices) and heterogeneous study protocols (design, comparator, observation period, tested body area) [58]. Furthermore, the authors recommended to standardize such issues, as they bias the value and results of the published QST–SCS trials and to explore novel, alternative SCS waveforms. Notably, in all studies it could not be excluded, that SCS exerted its effect on the contralateral non-painful extremity, hence interfering with QST measurements in a broader sense, while in contrast selective DRG stimulation may have the potential to overcome such limiting concerns [58].

With this in mind, HFS waveform (sub-perceptional/paresthesia-free) was compared to conventional tonic SCS and SCS “off” in a single-center trial including a broad variety of chronic pain disorders such as FBSS, migraine, neuritis and CRPS with SCS leads implanted at different spine levels (cervical—thoracic—occipital). Compared to tonic SCS and “off” SCS, HFS exhibited the most prominent effects by increasing mechanical detection thresholds, pressure pain and vibration detection thresholds suggesting a different pathway through which HFS may modulate the sensorimotor system [17, 58].

### Strengths and limitations

This is the first study exclusively assessing QST changes of a body area stimulated with the corresponding neural route (L4 DRG—CRPS of the knee) enrolling a homogenous cohort of CRPS patients. The effects observed in our study can be construed in relation to the stimulation of a selective, specific neural structure (L4 DRG) and we can exclude direct modulation of the contralateral, non-painful area as opposed to spinal cord stimulation. This study intentionally does not include a healthy control group. Rather, with each subject acting as their own control by comparing the diseased knee to the non-diseased within the same metabolic and genetic environment, we hope to demonstrate the changes in individuals using site-specific neurostimulation techniques: in this example, the DRG. Nevertheless, ongoing pathophysiological changes on the not-affected knee may be of relevance for the observed effects and shall be controlled using healthy subjects in further trials.

By utilizing the contralateral knee for comparison, the contrast in pre-stimulation neurophysiology to post-stimulation neurophysiology may be appreciated in this context. Compared to the study of Kemler and co-workers our observation period appears to be too short with a relatively low number of study participants.

First, there was a selection bias in the study as only those patients who passed the initial trial were included in the study (i.e., proceeded to a permanent implantation that allowed a 3-month follow-up). Second, there was no control of data collection bias such that the investigator who performed QST did not seem to be blinded to the patients' clinical treatment and conditions. In fact, it would be impossible to observe blindness in data collection given the patient selection bias. Third, there was no placebo control in this study. Another important issue with SCS and DRG stimulation that everyone recognizes but has been probably inadequately addressed in the literature is “tolerance” to the stimulation effect, which occurs at or shortly after 1 year. Hence, it has been mandated for clinical trials (SCS, DRG), to provide at least 1-year follow-up for outcome trials on SCS. However, current available neuromodulation sub-perceptional programming paradigms readily permit sham-control, comparative trials.

Given these facts, future neuromodulation in-human studies are recommended to increase evidence by including sham groups (placebo) and a standardized stimulation paradigm (electrode contact polarity, amplitude, frequency, intensity) [59]. However, several concerns have been identified related to sham-controlled interventional study protocols such as ethical, funding, and technical challenges in neuromodulation research [60].

Given these concerns and challenges, in-human studies combining different objective measure may considerable unveil mechanism and pathways relevant for CRPS and associated neural targets (dorsal root ganglion) and therefore potentially of value for future targeted neuromodulation research [61].

## Conclusions

Selective L4-DRGSTIM lowered ongoing pain in patients with persistent CRPS of the knee in parallel with improvements of emotional distress (depression). L4-DRG_STIM_ also evoked significant normalization in the pain domain of the somatosensory profile, which was associated with pain relief. Thermoreceptive and mechanoreceptive non-nociceptive perception remained unaffected. In aggregate, we conclude that QST is a valuable instrument to use to help identify DRG_STIM_ patients likely to respond well to long-term DRG therapy compared to those profile of patients, where DRG-stimulation efficacy remained weak or absent.

## Data Availability

The datasets used and/or analyzed during the current study are available from the corresponding author on reasonable request.

## Abbreviations

CDT: Cold detection threshold
WDT: Warm detection threshold
TSL: Thermal sensation threshold
CPT: Cold pain threshold
HPT: Heat pain threshold
PPT: Pressure pain threshold
MPT: Mechanical pain threshold
MP: Mechanical pain sensation
WUR: Wind-up ratio
CPT: Cold pain threshold
MDT: Mechanical detection threshold
VDT: Vibration detection threshold
DMA: Dynamic mechanical allodynia
PHS: Paradoxical heat sensation
IL: Interleukine
HMGB-1: High mobility group box
TNF: Tumor necrosis factor
